# Editorial: Untangle the broad connections and tight interactions between human microbiota and complex diseases through data-driven approaches

**DOI:** 10.3389/fmicb.2023.1157579

**Published:** 2023-02-15

**Authors:** Liang Wang, Zuo-Bin Zhu, Li Zhang, Jian Li, Qi Zhao

**Affiliations:** ^1^Laboratory Medicine, Guangdong Provincial People's Hospital (Guangdong Academy of Medical Sciences), Southern Medical University, Guangzhou, Guangdong, China; ^2^School of Medical and Health Sciences, Edith Cowan University, Perth, WA, Australia; ^3^School of Medical Informatics and Engineering, Xuzhou Medical University, Xuzhou, Jiangsu, China; ^4^Department of Genetics, School of Life Sciences, Xuzhou Medical University, Xuzhou, Jiangsu, China; ^5^School of Biotechnology and Biomolecular Sciences, University of New South Wales, Sydney, NSW, Australia; ^6^School of Public Health and Tropical Medicine, Tulane University, New Orleans, LA, United States; ^7^School of Computer Science and Software Engineering, University of Science and Technology Liaoning, Anshan, China

**Keywords:** microbiome, human diseases, metagenomics, metaomics, computational tools

It is well-known that microorganisms are ubiquitous in the environment and occupy almost all habitats in animals and humans (Finlay and Clarke, 1999; Rosenberg, 2021). Traditionally, microorganisms are studied as individuals grown in isolation under artificial conditions; however, with the development of experimental techniques and computational methods, microbes are now frequently considered as a functional group in a particular niche and studied at the community level in order to best mimicking the real-world situations (American Academy of Microbiology, 2004). During the study of microbial communities, two terms are commonly used, that is, microbiota and microbiome. A microbiota is defined as the microorganisms present in a defined environment and consists of bacteria, fungi, viruses, archaea, protists, etc. (Malard et al., 2021), while a microbiome not only means the collection of genomes from all the microorganisms in a niche, but also includes the microbial structural elements, metabolites, and the environmental conditions (Berg et al., 2020). For the past two decades, both sequencing technologies and mass spectrometry techniques have been developing rapidly. With their in-depth applications in the dissection of the human microbiota, more and more evidence have shown that microorganisms play very important roles in physiological functions and are closely related to various complex diseases in human beings (Hou et al., 2022). This has led to an insightful understanding of underlying disease mechanisms from microbial perspectives. Therefore, elucidation of the microbiota-disease association will be of great help for understanding the pathogenesis of human diseases, promoting early diagnosis, and improving precision medicine.

Particularly, in the human gut, the vast majority of gut microbes not only synthesize essential amino acids and vitamins but also facilitate the digestion of indigestible components of the human diet like plant polysaccharides (Rowland et al., 2017; Vernocchi et al., 2020). When gut microbial communities change, people are likely to suffer from related digestive system diseases, but when the abnormal gut microbial communities are restored to normality, disease symptoms could be alleviated (Gagliardi et al., 2018; Liu et al., 2021), though safety issues are still under intensive investigations (Daliri et al., 2018). If changes in intestinal microbes can be detected in time and given corresponding treatment, the workload of later clinical diagnosis and treatment will be greatly reduced (Zhang et al., 2015; Manor et al., 2020). Although current technologies have already helped us identify many previously unexpected connections between the microbiota and diseases, such as cancer, autoinflammatory diseases, metabolic syndromes, digestive system diseases, cardiovascular diseases, and central nervous system disorders, the present level of knowledge is still limited (Zhang et al., 2015). It is rather difficult to analyze the existing meta-omics data in-depth due to the lack of competent algorithms and bioinformatics tools, which leads to a narrow understanding of the microbiome-disease association and severely limits the development of the association mechanisms (Wang et al., 2022). Therefore, more efforts should be applied to the microbiota-disease association analysis, especially to promote the application of microbial analysis in the clinical settings for the diagnosis, treatment, and prevention of complex human diseases. In addition, downstream experimental validations of the microbiome discoveries in terms of the associations between microbiota and diseases are urgently needed to promote the real-world application of the meta-omics analysis. Therefore, studies with the combination of experimental and computational methods for interrogating the intriguing associations are also desirable.

In this Research Topic, all the collected articles could be divided into several groups that are either directly related to the topic by focusing on the interactions between microbiota and diseases or indirectly linked with the topic by focusing on models, tools, biomarkers and diseases, which are all summarized below to emphasize the core of the collection, that is, the broad connections and tight interactions between human microbiota and complex diseases. In specificity, Qin et al. compared the gut microbiome in 28 healthy people and 61 lung cancer patients that were classified into three types according to their histopathology, that is, Atypical Adenomatous Hyperplasia/Adenocarcinoma *in situ* (AAH/AIS), Minimally Invasive Adenocarcinoma (MIA), and Invasive Adenocarcinoma (IA). According to the results, categorized cancer patients had unique intestinal flora characteristics with comparatively lower density and flora diversity than healthy people. In addition, several flora markers were identified for the development of lung cancer, which held the potential for diagnosis, prognosis, prevention and treatment of lung cancer. Yang L. et al. from Sichuan University performed an integrative analysis of gut microbiota and fecal metabolites by comparing 32 metabolic associated fatty liver disease (MALFD) patients and 30 healthy individuals; according to the results, decreased species richness and diversity and altered β-diversity in feces were found in MALFD patients *via* 16S rRNA amplicon sequencing data, while metabolomic analysis identified overall changes in fecal and serum metabolites dominated by lipid molecules. Further associations between gut microbiota and fecal metabolites revealed that LPC 18:0 was positively correlated with *Christensenellaceae*_R-7_group, *Oscillospiraceae*_UCG-002 while neohesperidin was positively correlated with *Peptoniphilus, Phycicoccus*, and *Stomatobaculum*, which provided novel clues for understanding the molecular mechanisms of MALFD, and its diagnostic markers and therapeutic strategies. In addition, Wei et al. developed and validated an interpretable radiomic nomogram for severe radiation proctitis prediction in postoperative cervical cancer patients because radiation proctitis is a complex disease closely related to the microbiota but it is really time-consuming and expensive for analysis of gut microbiota. Therefore, this study emphasized the limitations of microbiota study and proposed a solution to solve the issue.

Except for the direct analysis of human microbiota and complex diseases, several studies focused on the constructions of models and development of tools for disease studies. For example, Yang B. et al. focused on the disease-ligand identification in the system of traditional Chinese medicine (TCM) based on a newly developed screening method termed as flexible neural tree (FNT) model, which were successfully applied to hypertension, diabetes, and COVID-19 for the identification of related compounds in TCM. It is also well-studied that hypertension, diabetes, and COVID-19 are closely associated with gut microbiota (Gurung et al., 2020; Mishima and Abe, 2021; Zhang F. et al., 2022). Therefore, the disease-ligand identification model has potential application in dissection the association between human microbiota and human diseases. In another study, Wang et al. tried to infer pan-cancer associated genes by examining the microbial model organism *Saccharomyces cerevisiae* by homology matching, which was based on the principle that the homologous genes of the common ancestor may have similarities in expression. According to the authors, their study holds the potential in revealing a link between microbiota and associated diseases, which is crucial to understand the molecular mechanisms of these diseases in the development of new microbiome-based therapies. In addition, Chen and Lei noticed that limitations of traditional medical experiments in the study of potential microbe-disease associations. Therefore, they proposed a method based on heterogeneous network and metapath aggregated graph neural network (MAGNN) to predict microbe-disease associations, which is termed as MATHNMDA. According to the results, their model could effectively predict microbe-disease associations in terms of case studies of asthma, inflammatory bowel disease, and COVID-19. In another study, Niu et al. studied an industrial yeast *Pichia pastoris* from the aspect of transcriptomic analysis, which aims to identify the regulation of foreign proteins with different stabilities expressed in *Pichia pastoris*. According to their results, the study shed a new light on the understanding of the regulatory mechanisms in yeast cells that responds to intracellular folding stress.

COVID-19 that is caused by the severe acute respiratory syndrome coronavirus 2 (SARS-CoV-2) has been widely spread worldwide since the end of 2019 (Liu et al., 2022; Zhang Y.-D. et al., 2022), which generated huge social and economic impact on human beings. Since COVID-19 infection is tightly associated with human microbiota, several studies also contributed to its diagnosis and therapy in this Research Topic collection. For example, Peng et al. developed a novel diagnostic analysis for CT scan images of COVID-19 pneumonia based on a deep ensemble framework with DenseNet, Swin transformer, and RegNet, which achieved the best precision of 0.9833, recall of 0.9895, accuracy of 0.9894, F1-score of 0.9864, AUC of 0.9991, and AUPR of 0.9986 under binary classification problem by comparing with other classification methods. Moreover, Tian et al. constructed a deep ensemble learning-based automated detection of COVID-19 using lung CT images and Vision Transformer and ConvNeXt, which computed the best precision of 0.9668, an accuracy of 0.9696, and an F1-score of 0.9631 in the three-classification experiment. In addition, Chen et al. built a novel weighted reconstruction-based linear label propagation (WLLP) algorithm for predicting potential therapeutic agents for COVID-19, which exhibited excellent performance with an AUC of 0.8828 ± 0.0037 and an area under the precision-recall curve of 0.5277 ± 0.0053, showing that the algorithm could be used to suggest potential drugs for the treatment of COVID-19.

Finally, there are also two studies that are focusing on microRNAs in human diseases. According to previous studies, it was well-known that the interactions between gut microbiota and microRNA affected host pathophysiology such as intestinal, neurological, cardiovascular, and immune health and diseases (Li et al., 2020). Therefore, it is meaningful to include a couple of microRNAs studies in this Research Topic. In particular, Qu et al. investigated the spring-like effect of microRNA-31 in balancing inflammatory and regenerative responses in colitis, according to which MIR31 is able to alleviate inflammation *via* inhibiting inflammatory cytokine receptors and can promote epithelial regeneration by modulating the WNT and Hippo signaling pathways. In the other study, Yao et al. identified circRNA-miRNA interactions based on multi-biological interaction fusion by proposing a novel model termed as circRNA-miRNA interaction prediction model (IIMCCMA), which showed that the model could achieve excellent performance in predicting the rare interaction between circRNA and miRNA, which helped to understand the molecular mechanism and contributed to the diagnosis, treatment, and prognosis of human diseases. However, whether these microRNAs involve any interactions with gut microbiota require further studies.

Taken together, a total of 12 articles including research papers, methodologies, web server tools, and software were enclosed in this Research Topic, which were authored by 96 investigators from different countries and regions of the whole world. The Research Topic focuses on human diseases such as cancer, pneumoniae, liver disease, colitis, and proctitis mainly from the aspects of human microbiota and relevant factors that could greatly facilitate the understanding of complex diseases in human beings from a long-term perspective. In addition, we would also like to thank all the reviewers for their valuable, rigorous, and high-standard suggestions and comments during the tedious peer review process. We would like to express our sincere gratitude to the Specialty Chief Editor, Dr. George Tsiamis, and also the editorial office of Frontier in Microbiology, for providing us with this opportunity to hold this fascinating Research Topic issue successfully.

## Author contributions

LW, Z-BZ, and QZ drafted the manuscript. All authors provided comments and feedbacks during the revision of the manuscript. All authors proposed the Research Topic theme, made a direct and intellectual contribution to the work, and approved the final version of the editorial for publication.
